# Classic Kaposi’s sarcoma - complete response to radiation therapy: a case report

**DOI:** 10.1186/s13256-016-1101-6

**Published:** 2016-11-10

**Authors:** Kennet Ramírez, José Zavala, David Morán, Diana Hernández, Alberto Jiménez

**Affiliations:** 1 Departments of Radiation Oncology, State Oncology Center, Avenida Lázaro Cárdenas número 208, esquina antiguo camino a Chiná, sector Las Flores. Código Postal, 24096 Campeche, México; 2 Departments of Pathology, State Oncology Center, Avenida Lázaro Cárdenas número 208, esquina antiguo camino a Chiná, sector Las Flores. Código Postal, 24096 Campeche, México

**Keywords:** Classic Kaposi’s sarcoma, Kaposi’s sarcoma, Palliative radiotherapy in classic Kaposi’s sarcoma, Case report

## Abstract

**Background:**

Classic Kaposi’s sarcoma is a lymphatic endothelial cell neoplasm usually present on the skin of the upper and lower extremities. Although it commonly affects human immunodeficiency virus positive patients, there have been some human immunodeficiency virus negative cases reported. We report an uncommon presentation of stage IV classic Kaposi’s sarcoma in an human immunodeficiency virus negative patient in Latin America with complete clinical response using only radiation therapy treatment.

**Case presentation:**

A 78-year-old Mexican man with no evidence of human immunodeficiency virus infection presented with a painful widespread dermatosis with maculopapular, nodular, violaceous lesions on his legs and ulcerated lesions on his feet. A biopsy confirmed the lesions as classic Kaposi’s sarcoma. Radiotherapy treatment was delivered, prescribing a total dose of 30 Gy in 15 fractions with a complete clinical response within 15 months of follow-up.

**Conclusions:**

This is an unusual case since it is uncommon to use radiation therapy as the single treatment in stage IV classic Kaposi’s sarcoma; the efficacy of the treatment is shown in the impact in our patient’s recurrence-free survival, local control, and palliation of our patient’s symptoms.

## Background

Kaposi’s sarcoma (KS) is a malignant neoplasm of lymphatic endothelial cells defined for the first time in 1872 [1]. A Hungarian dermatologist, Moritz Kaposi, named it as “idiopathic multiple pigmented sarcoma”, also known as “Kaposi’s angiosarcoma” or “idiopathic multiple hemorrhagic sarcoma”. KS is a systemic angiomatosis with malignant evolution, which is initially manifested as multiple vascular nodules in the skin and other organs [2, 3]. Classic Kaposi’s sarcoma (CKS) mostly affects people of Mediterranean background and Jewish origin, Italy and Turkey being the countries with the highest frequency of the disease. CKS appears between the fifth and seventh decade of life and is more common in males than females with a ratio of 15 to 1.

The most frequent location is the lower extremities with cutaneous affectation and centripetal extension. Tumors and nodules can either be covered by normal skin, atrophic skin or ulcerated; some others may have a wart-like or fungal surface. The lesions can be painful, edematous and hemorrhagic, limiting patient ambulation. The damage may appear on internal organs or be only visceral without any cutaneous lesions. Despite the fact that any internal organ can be affected, it can also appear without cutaneous lesions and exclusive visceral affection. The affection of the lungs or lymph nodes it is not frequent. Death can be a consequence of generalized disease with cachexia, hemorrhage, or impairment of vital organs function by tumor growth [4, 5].

The following table shows a classification of the most common forms found in the current literature, which divides CKS into four stages [6] (Table 1).

**Table 1:** Staging of classic Kaposi’s sarcoma

Lesions	Location	Behavior	Evolution	Complications
I Nodules/macules	Legs	Nonaggressive	A - slow B - fast	Lymphedema lymphorrhea
II Plaques	Legs	Locally aggressive	A - slow B - fast	Hemorrhage
III Angiomatous plaques and nodules	Limbs	Locally aggressive	A - slow B - fast	Functional damage
IV Angiomatous plaques and nodules	Limbs, trunk, head	Disseminated	B - fast	Ulceration

Stages I and II are subdivide into:

Group A: slow progression

Group B: rapid progression with an increased number of plates/nodules or the extension that the nodules occupy in 3 months since the last scan

Stages III and IV present more gastrointestinal and visceral affectation

## Case presentation

**Fig. 1:**
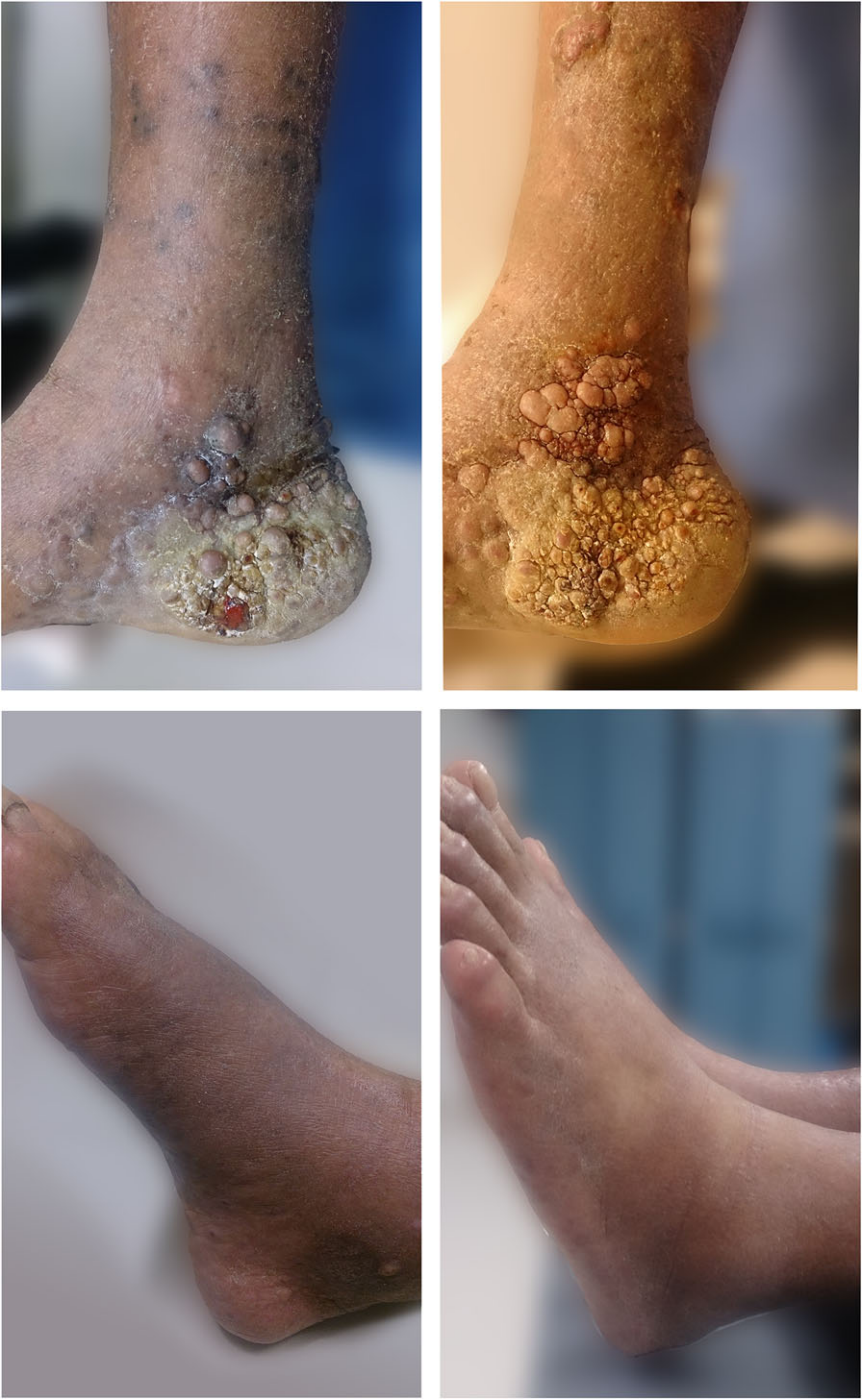
Cutaneous lesions affected the lower extremities in a symmetrical fashion, mainly heels and toes. Lesions reduced in size after treatment with radiation therapy. After 15 months of radiotherapy, a complete response was observed in his feet with improved mobility, complete disappearance of pain, and without significant toxicity

**Fig. 2:**
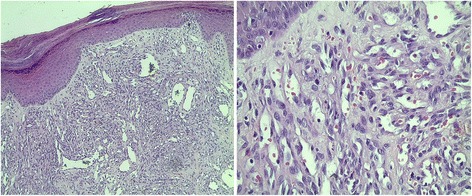
Kaposi’s sarcoma at low magnification. Kaposi’s sarcoma is mainly composed of spindle cells separated by vascular channels

**Fig. 3:**
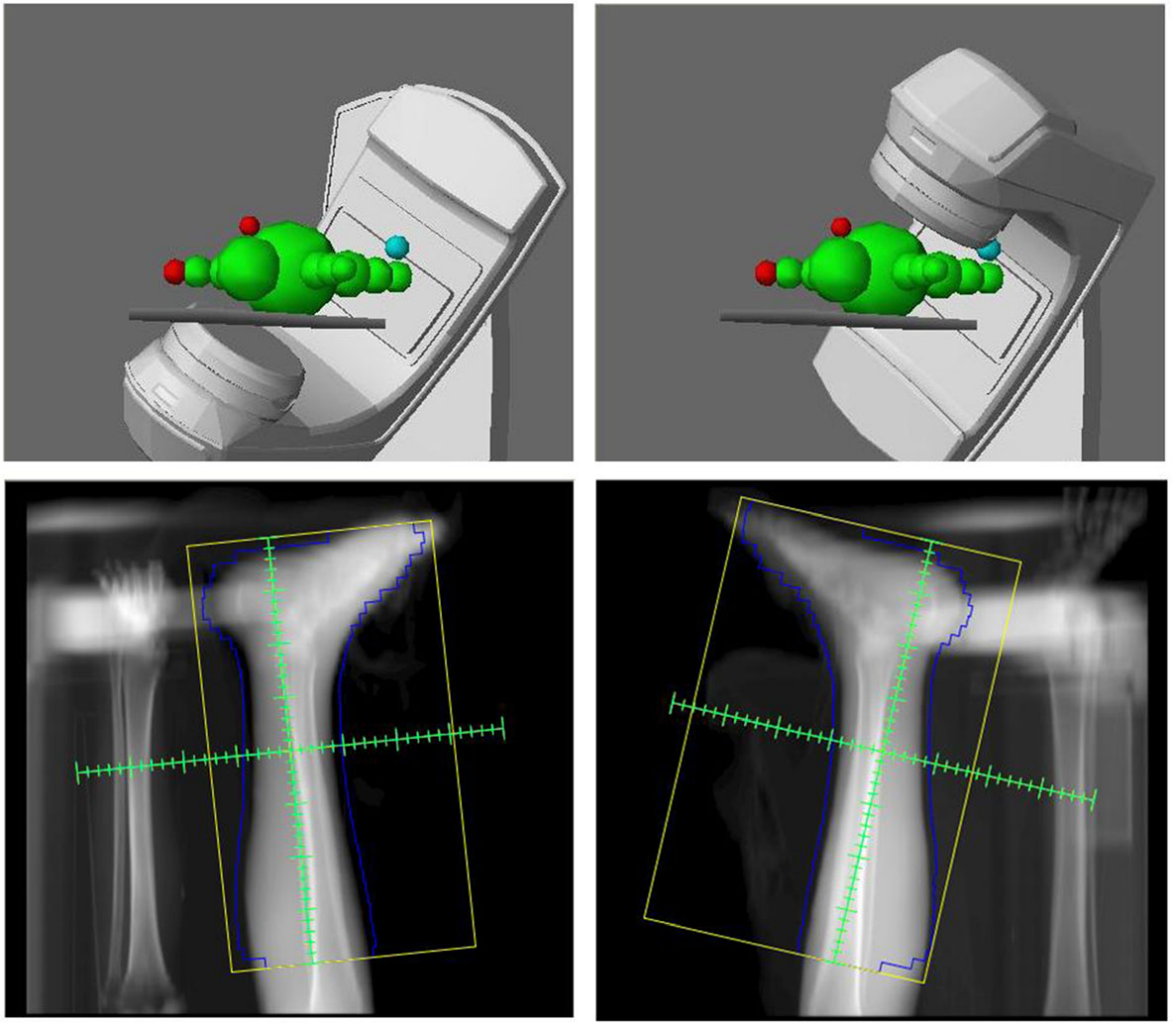
Field of treatment with Clinac iX equipment, two field photon mode

A 78-year-old Mexican man with no evidence of human immunodeficiency virus (HIV) infection presented with a history of two cardiac catheterizations, longstanding hypertension, high-risk prostate cancer treated with total androgenic blockage and radiation therapy (RT). Our patient also presented a painful widespread dermatosis with maculopapular, nodular, violaceous lesions on his legs and ulcerated lesions on his feet, limiting our patient’s ambulation for a year (Fig. 1). In order to confirm the diagnosis of CKS, an incisional biopsy was performed on our patient; the result was non-HIV-associated CKS without immunosuppression (Fig. 2). The final diagnosis was: dispersed and aggressive stage IV CKS. Our patient was not a candidate for systemic therapy due to the comorbidities and the high toxicity risk. Radiotherapy was decided as the single treatment with the Clinac iX energy 6MV equipment (Varian Medical Systems, Inc., Palo Alto, CA, USA), using two fields of treatment and 100 % of the prescription dose, giving a total dose of 3000 cGy in 15 fractions, 200 cGy per fraction, and a bolus of 0.5 cm doses to the surface. An increase in electrons for his heels with a 0.5 cm depth from the skin was considered (Fig. 3).

## Discussion

Treatment for classic Kaposi’s sarcoma is mainly palliative. Multidisciplinary treatment methods with different responses are applied to CKS therapy such as intralesional interferon alpha, cryotherapy, hormone therapy, laser removal, systemic chemotherapy, infrared coagulation and radiotherapy depending on the clinical form [7]. Radiotherapy is a useful method to improve the quality of life in patients with CKS and the response to treatment usually carries minimal toxicity [8]. CKS is generally considered sensitive to radiation therapy with good palliative and esthetic results, demonstrated with doses greater than 20 Gy [9]. In this sense in the literature, a standard dose of treatment is not mentioned; however, radiotherapy hypofractionation has demonstrated to have an impact in terms of recurrence-free survival, toxicity, and local control [10]. For those patients with a limited life expectancy one fraction with 800 cGy has shown to provide successful outcomes [11]. In this case, the dose of 30 Gy in sessions of 2 Gy per fraction in combination with electron therapy in the same dosage in the lesions had a complete response on his feet, along with the relief of symptoms and improvement in life quality of our patient.

## Conclusions

It is uncommon to use radiation therapy as the single treatment in stage IV classic Kaposi’s sarcoma, the efficacy of the treatment is shown in the impact in our patient’s recurrence-free survival, local control, and palliation of patient symptoms.

## Abbreviations

CKS: classic Kaposi’s sarcoma
HIV: Human Immunodeficiency Virus
KS: Kaposi’s sarcoma
RT: radiotherapy or radiation therapy

## References

[1] Szajerka T, Jablecki J. Kaposi’s sarcoma revisited. AIDS Rev. 2007;9:230–6. 18219366

[2] Sullivan RJ, Pantanowitz L, Casper C, Stebbing J, Dezube BJ. HIV/AIDS: epidemiology, pathophysiology, and treatment of Kaposi sarcoma-associated herpesvirus disease: Kaposi sarcoma, primary effusion lymphoma, and multicentric Castleman disease. Clin Infect Dis. 2008;47(9):1209–15. 10.1086/592298 PMC2700291 18808357

[3] Antman K, Chang Y. Medical progress: Kaposi’s sarcoma. N Engl J Med. 2000;342:1027–38. 10.1056/NEJM200004063421407 10749966

[4] Rojo DE. Armando: Acta Medica Grupo Angeles, Sarcoma de Kaposi; resumen 11, No 1 enero-marzo. 2013

[5] Iscovich J, Boffetta P, Winkelmann R, Brennan P. Classic Kaposi’s sarcoma as a second primary neoplasm. Int J Cancer. 1999;80:178–82. 10.1002/(sici)1097-0215(19990118)80:2<178::aid-ijc3>3.0.co;2-l 9935196

[6] Brambilla L, Boneschi V, Taglioni M, Ferrucci S. Staging of classic Kaposi’s sarcoma: a useful tool for therapeutic choices. Eur J Dermatol. 2003;13(1):83–6. 12609790

[7] Cooper JS, Steinfeld AD, Lerch I. Intentions and outcomes in the radiotherapeutic management of epidemic Kaposi’s sarcoma. Int J Radiat Oncol Biol Phys. 1991;20(3):419–22. 10.1016/0360-3016(91)90051-5 1995526

[8] Akmansu M, Goksel F, Erpolat OP, Unsal D, Karahacioglu E, Bora H. The palliative radiotherapy of classic Kaposi’s sarcoma of foot region: retrospective evaluation. Int J Hematol Oncol. 2014;24:147–52.

[9] Nicolini G, Abraham S, Fogliata A, Jordaan A, Clivio A, Vanetti E, Cozzi L. Critical appraisal of volumetric-modulated arc therapy compared with electrons for the radiotherapy of cutaneous Kaposi’s sarcoma of lower extremities with bone sparing. Br J Radiol. 2013;86:20120543. 10.1259/bjr.20120543 PMC3608058 23392192

[10] Singh NB, Lakier RH, Donde B. Hypofractionated radiation therapy in the treatment of epidemic Kaposi sarcoma--a prospective randomized trial. Radiother Oncol. 2008;88(2):211–6. 10.1016/j.radonc.2008.03.009 18439694

[11] Harrison M, Harrington KJ, Tomlinson DR, Stewart JS. Response and cosmetic outcome of two fractionation regimens for AIDS-related Kaposi’s sarcoma. Radiother Oncol. 1998;46(1):23–8. 10.1016/s0167-8140(97)00141-2 9488123

